# Brain Proteomic Profiling in Intractable Epilepsy Caused by TSC1 Truncating Mutations: A Small Sample Study

**DOI:** 10.3389/fneur.2020.00475

**Published:** 2020-05-29

**Authors:** Yi-Dan Liu, Meng-Yu Ma, Xi-Bin Hu, Huan Yan, Yan-Ke Zhang, Hao-Xiang Yang, Jing-Hui Feng, Lin Wang, Hao Zhang, Bin Zhang, Qiu-Bo Li, Jun-Chen Zhang, Qing-Xia Kong

**Affiliations:** ^1^Cheeloo College of Medicine, Shandong University, Jinan, China; ^2^Department of Imaging, Affiliated Hospital of Jining Medical University, Jining, China; ^3^Department of Neurology, Affiliated Hospital of Jining Medical University, Jining, China; ^4^Clinical Medical College, Jining Medical University, Jining, China; ^5^Department of Neurosurgery, Affiliated Hospital of Jining Medical University, Jining, China; ^6^Department of Pediatrics, Affiliated Hospital of Jining Medical University, Jining, China; ^7^Institute of Epilepsy, Jining Medical University, Jining, China

**Keywords:** tuberous sclerosis complex, TSC1 gene, epilepsy, proteomic profiling, mutation

## Abstract

Tuberous sclerosis complex (TSC) is a genetic disease characterized by seizures, mental deficiency, and abnormalities of the skin, brain, kidney, heart, and lungs. TSC is inherited in an autosomal dominant manner and is caused by variations in either the TSC1 or TSC2 gene. TSC-related epilepsy (TRE) is the most prevalent and challenging clinical feature of TSC, and more than half of the patients have refractory epilepsy. In clinical practice, we found several patients of intractable epilepsy caused by TSC1 truncating mutations. To study the changes of protein expression in the brain, three cases of diseased brain tissue with TSC1 truncating mutation resected in intractable epilepsy operations and three cases of control brain tissue resected in craniocerebral trauma operations were collected to perform protein spectrum detection, and then the data-independent acquisition (DIA) workflow was used to analyze differentially expressed proteins. As a result, there were 55 up- and 55 down-regulated proteins found in the damaged brain tissue with TSC1 mutation compared to the control. Further bioinformatics analysis revealed that the differentially expressed proteins were mainly concentrated in the synaptic membrane between the patients with TSC and the control. Additionally, TSC1 truncating mutations may affect the pathway of amino acid metabolism. Our study provides a new idea to explore the brain damage mechanism caused by TSC1 mutations.

## Introduction

Epilepsy is one of the most common neurological diseases in the clinic. It is a clinical syndrome caused by abnormal discharge of highly synchronized neurons in the brain caused by a variety of reasons, accompanying psychiatric and neurological comorbidities (1). The etiological mechanism of epilepsy is very complex, and genetic, metabolic, and structural factors are important causes of epilepsy (2). Tuberous sclerosis complex (TSC) is a typical neurocutaneous syndrome characterized by cerebral and dermatological lesions, with hamartoma formation in multiple organ systems (3). TSC is typically characterized by severe neurodevelopmental disorders, which generally include mental disabilities, autism, and other behavioral and psychiatric symptoms. Therefore, these neurodevelopmental impairments were named TSC-associated neuropsychiatric disorders (TAND) (4). TSC-related epilepsy (TRE) is the most prevalent and challenging clinical manifestation of TSC (5). More than 70–80% of individuals with TSC have a history of seizures, which are often refractory to treatment, even with multiple antiepileptic drugs (6).

TSC is an autosomal dominant inherited disease. Most TSC patients are caused by mutations in the TSC1 or TSC2 genes that encode two proteins, hamartin (TSC1) and tuberin (TSC2). Under normal circumstances, these two proteins bind together to form a protein dimer complex that can inhibit the mammalian target rapamycin (mTOR) protein complex. The mTOR pathway is a crucial cellular signaling hub that integrates cell growth, proliferation, metabolism, and protein synthesis (7). TSC1 or TSC2 mutations cause excessive activation of mTOR pathway, leading to the promotion of cell growth and proliferation that caused a wide variety of tumor cell growth in TSC, including renal angiomyolipoma (RAML), pulmonary lymphangioleiomyomatosis (PLAM), subependymal giant cell astrocytoma (SEGA) and facial angiofibroma (8). The mTOR inhibitors, such as everolimus and rapamycin, are effective to treat tumors of TSC, however, their effect on the neurological symptoms is limited. Although everolimus therapy is likely to be effective for focal-onset seizures in TSC, most patients still have seizures, and many benefit little from the treatment (9).

Typical pathological of brain damage in TSC is cortical tubers, which are focal malformations of the cerebral cortex, marked by localized cortical destruction and varied abnormal cells, including deformed neurons, astrocyte proliferation, and giant cells (10). A few studies showed that autism may be associated with tubers in the temporal lobes (11), but the correlation between TAND and tubers is controversial (12). A growing body of evidences suggested that autism and cognitive deficit are more closely related to factors independent of tubers, like underconnectivity across multiple white matter fiber bundles (13). It is a remarkable fact that the brain damage caused by cortical tubers has generally been thought to contribute to epileptic seizures in TSC, for surgical removal of epileptogenic tubers can eliminate seizures to a great extent in some TSC patients (14). Additionally, when the treatment of the first two antiepileptic drugs (AEDs) are failed, adding new anti-epileptic drugs is generally not effective. Therefore, surgery is a good choice for patients who can undergo focal resection, and it may improve the outcome in future cohorts (15).

There are still many puzzles in the linkage from genetic mutations to cortical tubers, to infantile seizures, and late childhood mental retardation in TSC. Some studies have shown that the existence of TSC2 inactivation variants may affect the severity of TSC-related brain damage, and children with TSC2 protein truncation mutations are more likely to develop progressive cognitive deterioration and severe intellectual disability (16). The truncating mutation is the commonest mutation type in the TSC1 (80%) and the TSC2 (65%) genes. However, the clinical studies about brain damage related to TSC1 truncating mutations are rarer, due to TSC1 mutations account for the minority of all TSC patients as compared to TSC2 mutations, with the ratio of 1:5 (TSC1/TSC2) (17). In clinical practice, we found several patients of intractable epilepsy caused by TSC1 truncating mutations. To study the changes of protein expression in the damaged brain tissue with TSC1 truncating mutation, the protein spectrum detection was performed.

In this study, we reported clinical features, mutation analyses and brain proteomic profiling of three surgical cases of intractable epilepsy caused by truncating mutations in TSC1.

## Materials and Methods

We analyzed the clinical data and genetic testing of patients with TSC treated in the Multidisciplinary Center for Epilepsy of Affiliated Hospital of Jining Medical University from August 2018 to August 2019 and found three surgical cases of intractable epilepsy with truncating mutations in TSC1. To explore the difference in the expression of proteins between brain tissues with TSC1 truncating mutations and control brain tissues, proteomic profiling was performed.

### Clinical Data Collection

Basic information and medical history including age, gender, age of onset, seizure form and medication history were collected. Inclusion criteria: meeting the latest revised diagnostic criteria for TSC in 2012 (18). Exclusion criteria: brain lesions and seizures caused by other diseases; TSC complicated with other brain injuries, such as cerebrovascular disease, brain trauma, and encephalitis.

Video-electroencephalogram (VEEG): Scalp electrodes were placed, and VEEG monitoring was conducted. Then EEG signals were recorded by the Nicolet One VEEG analysis system (Nicolet, inc., USA). Magnetic resonance imaging (MRI): The 3.0 T magnetic resonance system (Siemens, Germany) was used to scan the transverse, coronal and sagittal images at 1 mm per layer. MRI morphological analysis program (MAP) and PET-MR image fusion was used to assist in locating epileptogenic lesions. Surgery and pathology: Electrophysiological monitoring was performed in surgery. Epileptogenic tubers and related cortex were completely removed under the premise of no disruption in brain function. After surgery, HE staining was performed to observe the morphological structure of brain tissues in a light microscope. IQ test: The intelligence of patients aged 6 to 16 was measured by the Wechsler Intelligence Scale for Children (5th ed.).

### Gene Detection and Bioinformatic Analysis

Peripheral blood samples were collected from patients and their families for gene detection. Next-generation sequencing (NGS) and multiplex ligation-dependent probe amplification (MLPA) were used to detect the variations in the TSC1 and TSC2 genes. The candidate mutation sites were then verified by Sanger sequencing.

#### Next-Generation Sequencing (NGS)

The genomic DNA was extracted from the blood samples by the FlexiGene DNA kit (Qiagen, Duesseldorf, Germany). The PrimeStar HS DNA Polymerase (New England BioLabs, Inc.) was used to amplify the DNA library. The SureSelect Target Enrichment System (Agilent Technologies, Inc.) was used to capture the target DNA fragments. Single-read sequencing was performed using NextSeq500 (Illumina, San Diego, USA). Sequences were aligned to UCSC hg19 by Burrow-Wheeler Aligner Version 0.7.15. Then single-nucleotide and deletion/insertion polymorphisms analyses were implemented to get mutation information in the targeted regions by GATK Version 3.6. According to the American College of Medical Genetics and Genomics (ACMG) criteria and guidelines, mutations were classified into five levels: “pathogenic,” “likely pathogenic,” “benign,” “likely benign,” and “uncertain significance” (19).

#### Multiplex Ligation-Dependent Probe Amplification (MLPA)

Microdeletions or microduplications in the TSC1 and TSC2 genes were detected by MLPA, using the SALSA^®^ MLPA^®^ Probemix P124-C3 TSC1 and P337-B1 TSC2 (MRC, Holland) as per the manufacturer's protocol. Capillary electrophoresis was implemented using ABI3130 DNA Sequencer (Applied Biosystems, USA). The software Coffalyser was subsequently applied to analyze the data.

#### Sanger Sequencing

The candidate mutated regions were selected for further validation to exclude false-positive sites. The primers were designed by PrimerZ^1^. The candidate variants were amplified by PCR using an EasyTaq PCR SuperMix (TransGen Biotech, China) (20). PCR products were then sequenced by Sanger sequencing.

#### Bioinformatic Analysis

To investigate the effects of the detected mutations on protein structure, we used the online tool PSIPRED V4.0^2^ to predict the secondary structures of the mutated and wild-type TSC1 proteins (21).

### Proteomic Profiling in Human Brain Tissue

To study the proteomic changes in the brain, the above three cases of diseased brain tissue with TSC1 truncating mutations resected in intractable epilepsy operations and the other three cases of control brain tissue resected in craniocerebral trauma operations were collected (the information of the control group shown in the Supplementary Table 1). Before the protein spectrum detection, genomic DNA was extracted from the six brain tissues and whole exome sequencing (WES) was performed. In the three patients with TSC, the results of WES in the brain were consistent with those of gene detection in peripheral blood. And there was no pathogenic mutation detected in the three control brain tissues. Then, the six brain samples were divided into the TSC1 truncating group and the control group for protein spectrum detection.

#### Experimental Design and Statistical Rationale

Two group samples were selected, and the group is represented by 3 biological replicates. For library generation by DDA, all 6 samples were pooled as a mixture and fractionated by high pH separation with 10 fractions. And all 6 samples were processed by data-independent acquisition (DIA) individually to assess the proteome differences. MS1 and MS2 data were all acquired, and samples acquisition by random order. The statistical analysis of the DIA dataset was performed by Spectronaut 13 (Biognosys AG, Switzerland) including data normalization and relative protein quantification. After Welch's ANOVA Test, differently expressed proteins were filtered with a *P* < 0.05 and fold change of more than 1.5.

#### Sample Pretreatment for Mass Spectrometric Analysis

The brain samples were treated by lysis buffer, which contains 1% SDS, 8 M urea, and Protease Inhibitor Cocktail (Roche Ltd. Basel, Switzerland), and then were put on ice for 30 min. The samples were centrifuged at 15,000 rpm for another 15 min at 4°C, and the supernatant was collected. After quantifying total protein concentration, a total protein of 100 μg per sample was transferred to a new tube with the final volume adjusted to 100 μL by the addition of 8M urea. The tris (2-carboxyethyl) phosphine (TCEP) was added to reduce the extracted proteins and then incubated at 37°C for 1 h. Alkylation of the samples was carried out as the manufacturer's protocol. After that, the proteins were digested by trypsin (Promega, Madison, WI) at 37°C overnight. The C18 ZipTip was used to desalt the digested peptides. Then, the Pierce™ Quantitative Colorimetric Peptide Assay (23275) and the SpeedVac were used in quantification and lyophilization, respectively (22).

#### Data-Independent Acquisition (DIA)

The peptide mixture was re-dissolved in the buffer A, which containing 20 mM ammonium formate in water adjusted to pH 10.0 with ammonium hydroxide, and then fractionated by high pH fractions using an Ultimate 3000 system (ThermoFisher Scientific, MA, USA) connected to a reverse-phase column (XBridge C18 column, Waters Corporation, MA, USA). Ten fractions were collected and each fraction was dried in a vacuum concentrator. The samples were re-dissolved in the solvent A containing 0.1% formic acid and then LC-MS/MS was acquired on an Orbitrap Lumos coupled to EASY-nLC 1200 system (Thermo Fisher Scientific, MA, USA). 3 μL of each peptide samples were loaded onto an analytical column (Acclaim PepMap C18, Thermo Fisher Scientific, MA, USA) and separated with a 130-min gradient from 4 to 50% B (0.1% formic acid in ACN). The mass spectrometer was worked under data-dependent acquisition (DDA) mode, and could automatically switch between MS and MS/MS mode. The parameters were set as (1) MS: scan range (m/z) of 350–1500; resolution of 60,000; automatic gain control (AGC) target value of 4e5; maximum injection time of 50 ms; dynamic exclusion of 30 s; (2) HCD-MS/MS: the resolution of 30,000; AGC target value of 5e4; maximum injection time of 86 ms; collision energy of 30.

#### Data Analysis

We use the Spectronaut 13 (Biognosys AG, Switzerland) to process and analyze the raw data of DIA with default settings to obtain the initial target list, which contained 136,450 precursors, 109,674 peptides, 8,399 proteins, and 8,316 protein groups. Spectronaut was installed to search the database of homo_sapiens_sp_201907.fasta assuming trypsin as the digestion enzyme. Carbamidomethyl (C) and Oxidation (M) were employed as the fixed modification and the variable modification, respectively. The false discovery rate (FDR) reflected by Q value on precursor and protein level was applied at 1%. The major group quantities were calculated by the average top 3 filtered peptides which passed the 1% Q value cutoff. After Welch's ANOVA Test, differently expressed proteins were filtered with a *P* < 0.05 and fold change of more than 1.5.

#### Bioinformatics Analysis

The volcano plot, a type of scatter-plot that is used to quickly identify changes in large data sets composed of replicate data, is drawn using the ggplot2 package^3^ Blast2GO version 5 and GOATOOLS were used for functional annotation and GO enrichment analysis, respectively.

## Results

### Clinical Data

**Figure 1:**
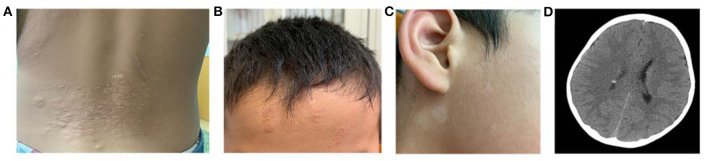
Clinical manifestations of TSC. **(A)** Shagreen patch at the waist of case 1. **(B)** Ffibrous cephalic plaque of case 2. **(C)** Hypomelanotic macule in the face of case 2. **(D)** A nodular dense shadow in the lateral wall of the right lateral ventricle in cranial CT of case 2, considered as a subependymal nodule.

All three cases were male and had their first seizure at the age of 3, 5, and 20, respectively, presenting as complex partial seizures (Table 1). Taking two or more AEDs, seizures were not well-controlled. All three had typical skin manifestations and subependymal nodules (Figure 1). Besides, case 2 had cardiac rhabdomyoma and multiple renal cysts.

**Table 1:** Epilepsy history of the three patients.

	**Age (year)**	**Gender**	**Age of onset (year)**	**First seizure form**	**Medication**	**Seizure frequency before surgery**
Case 1	10	Male	3	Complex partial seizure	Rapamycin, Oxcarbazepine, Sodium valproate, Vigabatrin	1–2 times a day
Case 2	6	Male	5	Complex partial seizure	Sodium valproate, Carbamazepine	4–5 times a day
Case 3	25	Male	20	Complex partial seizure	Sodium valproate, Oxcarbazepine	2–3 times a month

**Figure 2:**
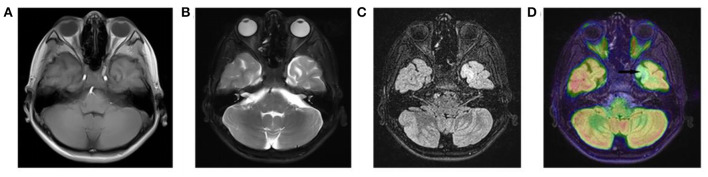
The Cranial MRI and PET-MR image fusion of case 1. There are **(A)** mixed T1WI signals, **(B)** long T2WI signals and **(C)** long T2-FLAIR signals in the left temporal lobe. **(D)** PET-MR image fusion shows decreased metabolism (arrow) in the corresponding positions.

**Figure 3:**
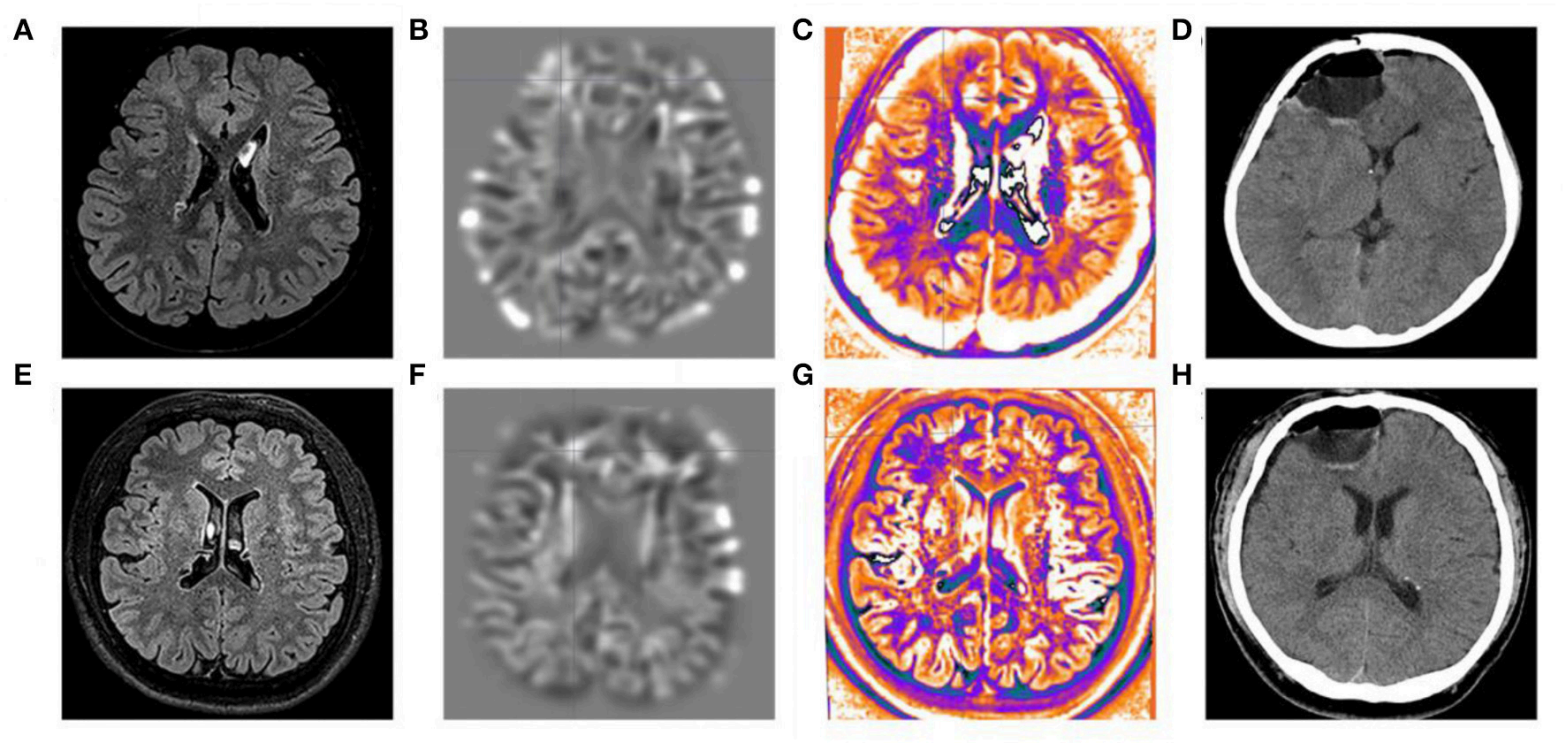
MRI morphological analysis and postoperative CT of case 2 and case 3. The **(A)** FLAIR, **(B)** junction imaging, **(C)** normalized FLAIR signal intensity (NFSI), and **(D)** postoperative CT of case 2. The **(E)** FLAIR, **(F)** junction imaging, **(G)** NFSI, and **(H)** postoperative CT of case 3.

**Figure 4:**
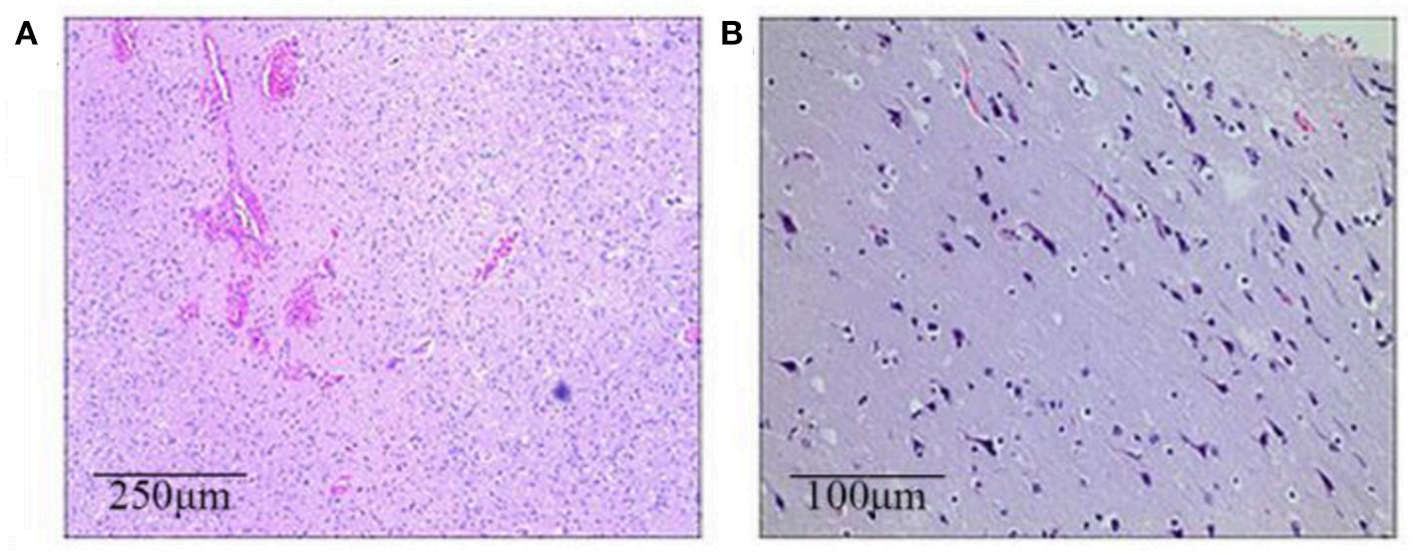
The brain histopathological results (HE staining). **(A)** Case 1 (the left frontotemporal lobe): Immature neurons, proliferated glial cells, and proliferated vessels with dilation and hyperemia can be seen in a light microscope. **(B)** Case 2 (the right frontal lobe): Neuronal shrinkage and degeneration can be seen, with the appearance of immature neurons and mild proliferation of glial cells.

All three patients had undergone epilepsy surgery. Take case 1 for example, VEEG showed that there were medium-high amplitude sharp waves and spike-and-wave complexes in the left frontal, middle, and temporal regions (Supplementary Figure 1). Cranial MRI showed abnormal signals in left frontal and temporal lobes, and PET-MR image fusion indicated that metabolism decreased in epileptogenic lesions (Figure 2). Combined with VEEG and imaging results, epileptogenic lesions were considered to be located in the left frontotemporal lobe. Epileptogenic tubers and related cortex were completely removed under the premise of no disruption in brain function. We also used the MRI morphological analysis program (MAP) to locate epileptogenic lesions, and the lesions of case 2 and case 3 were both located in the right frontal lobe (Figure 3). After surgery, HE staining was performed and the morphological structure of brain tissues was observed under the light microscope. The result indicated cortical dysplasias (Figure 4).

The patients were followed up for more than 8 months. The complete seizure freedom (Engel I) was achieved in case 1 and case 2. Case 3 had twice epileptic episodes within 1 month after surgery for the consecutive night shift, and then there was no seizure for 10 months (Engel II). Additionally, cases 1 and 2 assessed intelligence on the Wechsler Intelligence Scale for Children (5th ed.). The IQ of case 1 (10 years old) was 60, and he dropped out of school because of his frequent seizures and returned to school after the surgery. The IQ of case 2 (6 years old) was 80, and he was introverted and slow to study new things. Also, case 3 did not come to the hospital for an IQ test, and through telephone follow-up, we learned that he went to work normally after the operation.

### Genetic Findings

NGS found that all three cases had heterozygous mutations in the TSC1 gene, and Sanger sequencing was used to verify the authenticity and origin of the variation (Table 2, Supplementary Figure 2). Case 1 had the heterozygous mutation c.1524C>A, which was *de novo*. Case 2 had the heterozygous mutation c.1525C>T inherited from the mother. Case 3 had the heterozygous mutation c.1004delC inherited from the father. No TSC2 mutation was found in all three patients.

**Table 2:** Mutations in the TSC1 gene.

	**Mutation site (NM_000368)**	**Mutation type**	**Amino acid change**	**ACMG level**	**Mutation origin**
Case 1	c.1524C>A	Non-sense mutation	p.Tyr508Ter	Pathogenic	*De novo*
Case 2	c.1525C>T	Non-sense mutation	p.Arg509Ter	Pathogenic	Mother
Case 3	c.1004delC	Frameshift mutation	p.Thr335AsnfsTer3	Likely pathogenic	Father

**Figure 5:**
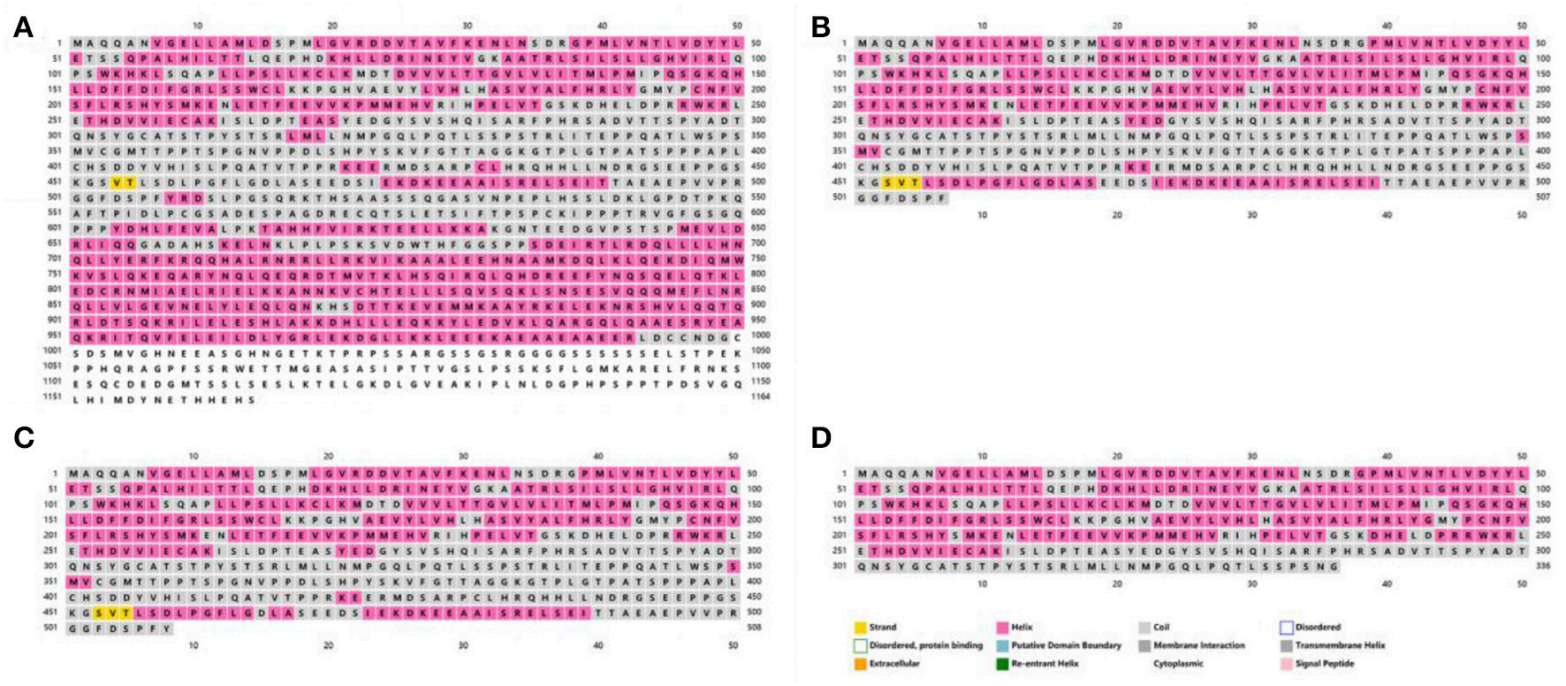
The secondary structures of the mutated and wild-type TSC1 proteins predicted by PSIPRED V4.0. The amino acid sequences and secondary structures of **(A)** wild-type, **(B)** case 1 (c.1524C>A, p.Tyr508Ter), **(C)** case 2 (c.1525C>T, p.Arg509Ter) and **(D)** case 3 (c.1004delC, p.Thr335AsnfsTer3).

To observe the effects of the detected variants on protein structure, we used PSIPRED V4.0 to forecast the mutated and wild-type TSC1 protein structures (Figure 5). The wild-type TSC1 protein has 1164 amino acids, and the mutation c.1524C>A and the mutation c.1525C>T are nonsense mutations, resulting in the absence of 657 amino acids and 656 amino acids, respectively. The mutation c.1004delC is a frameshift mutation that causes the amino acid changes starting at position 335, while the amino acid at position 337 is converted to a stop codon. This mutation resulted in the premature termination of protein synthesis, resulting in a protein deletion of 828 amino acids. All three mutations can result in premature termination of protein synthesis, called truncating mutations.

There were no microdeletions or microduplications in the TSC1 and TSC2 genes of the three cases detected by MLPA (data not shown).

### Proteomic Profiling

**Figure 6:**
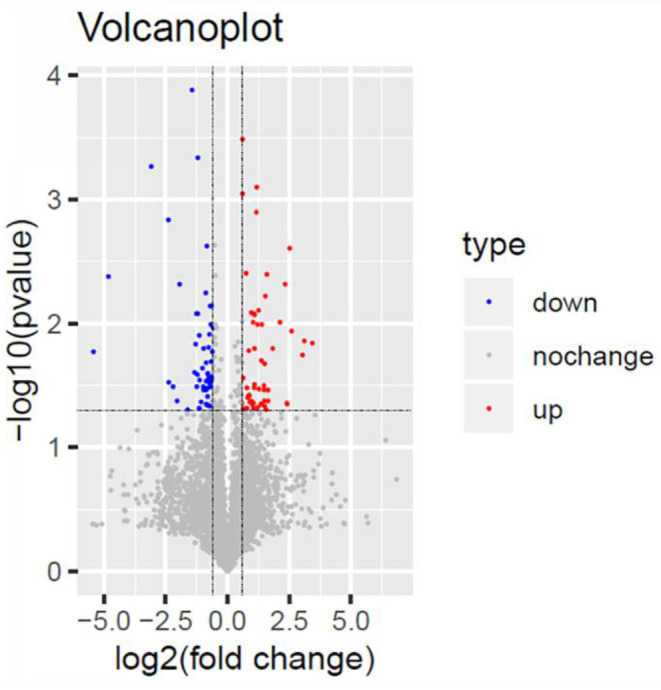
Volcano Plot. Volcano Plot of the 110 significantly dysregulated proteins between the TSC1 truncating mutation group and the control group. Blue represents the downregulated protein expression and red indicates the upregulated protein.

We analyzed the differentially expressed protein expression between the TSC1 truncating mutation group and the control group based on DIA and further studied the interaction between the detected differentially expressed proteins and their biological signal pathways by bioinformatics techniques, to explore whether there are other potential pathways in patients with TSC1 mutations. A total of 110 differentially expressed proteins were identified by proteomic analysis, and the fold change of these proteins was >1.5 (fold change> 1.5), and the differences were significant (*p* < 0.05). Among them, 55 proteins were up-regulated and 55 proteins were down-regulated (Table 3, Figure 6). The minimum fold change, of 1.506 was observed for ubiquitin-like modifier-activating enzyme 5 (UBA5), ranging up to >43.20-fold change for Complement component C8 alpha chain (C8A). Specifically, the folding changes of these proteins ranged from 1.5 to 43.2. Among them, 97 (88.18%) proteins fold varied between 1.5 and 5, 10 (9.09%) proteins fold varied between 5 and 10 times, and 3 (2.73%) proteins fold varied between 10 and 50 times.

**Table 3:** Differentially expressed proteins in the TSC1 truncating mutation group and the control group.

**Accession**	**Description**	**Anova (p)**	**Fold Change**	**Localization**
P07357	Complement component C8 alpha chain (C8A)	0.016983611	−43.2	Secreted/Cell membrane
P03952	Plasma kallikrein (KLKB1)	0.004165552	−28.29	Secreted
O75509	Tumor necrosis factor receptor superfamily member 21 (TNFRSF21)	0.00053877	−8.54	Cell membrane
Q86TI0	TBC1 domain family member 1 (TBC1D1)	0.001475815	−5.29	Nucleus
O43684	Mitotic checkpoint protein BUB3 (BUB3)	0.030033518	−5.19	Nucleus
Q9ULF5	Zinc transporter ZIP10 (SLC39A10)	0.032791468	−4.62	Membrane
P78347	General transcription factor II-I (GTF2I)	0.042513627	−4.14	Cytoplasm/Nucleus
A0A075B6K5	Immunoglobulin lambda variable 3–9 (IGLV3–9)	0.004807315	−3.84	Secreted/Cell membrane
P20851	C4b-binding protein beta chain (C4BPB)	0.000132275	−2.71	Secreted
Q9Y5K3	Choline-phosphate cytidylyltransferase B (PCYT1B)	0.025181613	−2.52	Endoplasmic reticulum
Q9HBF4	Zinc finger FYVE domain-containing protein 1 (ZFYVE1)	0.014720448	−2.44	Golgi apparatus/Endoplasmic reticulum
P22304	Iduronate 2-sulfatase (IDS)	0.008295384	−2.4	Lysosome
P24310	Cytochrome c oxidase subunit 7A1, mitochondrial (COX7A1)	0.032465751	−2.38	Mitochondrion inner membrane
Q92743	Serine protease HTRA1 (HTRA1)	0.025910263	−2.33	Cell membrane/Secreted/Cytoplasm
Q9Y673	Dolichyl-phosphate beta-glucosyltransferase (ALG5)	0.00833106	−2.32	Endoplasmic reticulum membrane
P02008	Hemoglobin subunit zeta (HBZ)	0.000461784	−2.3	
Q92574	Hamartin (TSC1)	0.048987683	−2.23	Cytoplasm/Membrane
Q8N135	Leucine-rich repeat LGI family member 4 (LGI4)	0.012625385	−2.22	Secreted
Q02880	DNA topoisomerase 2-beta (TOP2B)	0.028995164	−2.2	Cytoplasm/Nucleus
P35749	Myosin−11 (MYH11)	0.04901692	−2.18	Melanosome
Q03169	Tumor necrosis factor alpha-induced protein 2 (TNFAIP2)	0.043178123	−2.11	
P41218	Myeloid cell nuclear differentiation antigen (MNDA)	0.023166305	−2.02	Nucleus/Cytoplasm
Q12767	Transmembrane protein 94 (TMEM94)	0.032776619	−1.99	Membrane
Q8N4V1	Membrane magnesium transporter 1 (MMGT1)	0.034744035	−1.97	Endoplasmic reticulum membrane/Early endosome membrane
Q6X4W1	NMDA receptor synaptonuclear signaling and neuronal migration factor (NSMF)	0.015904018	−1.96	Nucleus/Cytoplas
O15027	Protein transport protein Sec16A (SEC16A)	0.00570868	−1.85	Endoplasmic reticulum membrane/Golgi apparatus membrane/Cytoplasm/ Microsome membrane
O60241	Adhesion G protein-coupled receptor B2 (ADGRB2)	0.020675302	−1.83	Cell membrane
Q13303	Voltage-gated potassium channel subunit beta−2 (KCNAB2)	0.044603556	−1.83	Cytoplasm/Membrane
P61923	Coatomer subunit zeta−1 (COPZ1)	0.029204203	−1.83	Cytoplasm/Golgi apparatus membrane
Q9H469	F-box/LRR-repeat protein 15 (FBXL15)	0.034625071	−1.82	Cytoplasm
Q9HB21	Pleckstrin homology domain-containing family A member 1 (PLEKHA1)	0.00236854	−1.79	Cytoplasm/Cell membrane/Nucleus
Q9NP77	RNA polymerase II subunit A C-terminal domain phosphatase SSU72 (SSU72)	0.045603854	−1.77	Nucleus/Cytoplasm
P21453	Sphingosine 1-phosphate receptor 1 (S1PR1)	0.025377411	−1.76	Cell membrane/Endosome
P20042	Eukaryotic translation initiation factor 2 subunit 2 (EIF2S2)	0.039044968	−1.75	
P30048	Thioredoxin-dependent peroxide reductase, mitochondrial (PRDX3)	0.033305951	−1.74	Mitochondrion/Cytoplasm/Early endosome
O75077	Disintegrin and metalloproteinase domain-containing protein 23 (ADAM23)	0.025278943	−1.74	Cell membrane/Secreted
P16389	Potassium voltage-gated channel subfamily A member 2 (KCNA2)	0.045913807	−1.71	Cell membrane/Cell projection/Cell junction
Q96D05	Uncharacterized protein FAM241B (FAM241B)	0.027804687	−1.7	Membrane
Q99707	Methionine synthase (MTR)	0.01578569	−1.7	Cytoplasm
O94817	Ubiquitin-like protein ATG12 (ATG12)	0.012333544	−1.66	Cytoplasm/Preautophagosomal structure membrane
Q9Y6D5	Brefeldin A-inhibited guanine nucleotide-exchange protein 2 (ARFGEF2)	0.030295761	−1.65	Cytoplasm/Membrane/Golgi apparatus
Q9BZL4	Protein phosphatase 1 regulatory subunit 12C (PPP1R12C)	0.04669813	−1.64	Cytoplasm
Q9Y6N8	Cadherin−10 (CDH10)	0.007311272	−1.63	Cell membrane
Q7L592	Protein arginine methyltransferase NDUFAF7, mitochondrial (NDUFAF7)	0.028619986	−1.62	Mitochondrion
O60256	Phosphoribosyl pyrophosphate synthase-associated protein 2 (PRPSAP2)	0.032180434	−1.6	
Q96CS3	FAS-associated factor 2 (FAF2)	0.01013332	−1.59	Cytoplasm/Endoplasmic reticulum
O60831	PRA1 family protein 2 (PRAF2)	0.029608992	−1.58	Endosome membrane
P62318	Small nuclear ribonucleoprotein Sm D3 (SNRPD3)	0.020213847	−1.58	Cytoplasm/Nucleus
Q8NBM8	Prenylcysteine oxidase-like (PCYOX1L)	0.046823812	−1.58	Secreted
Q15173	Serine/threonine-protein phosphatase 2A 56 kDa regulatory subunit beta isoform (PPP2R5B)	0.027312094	−1.57	Cytoplasm
Q08170	Serine/arginine-rich splicing factor 4 (SRSF4)	0.007248362	−1.57	Nucleus speckle
O75420	GRB10-interacting GYF protein 1 (GIGYF1)	0.027976441	−1.56	
P49458	Signal recognition particle 9 kDa protein (SRP9)	0.016962845	−1.54	Cytoplasm
P68366	Tubulin alpha−4A chain (TUBA4A)	0.042051132	−1.51	Cytoplasm
Q9GZZ9	Ubiquitin-like modifier-activating enzyme 5 (UBA5)	0.010959137	−1.51	Cytoplasm/Nucleus/Golgi apparatus
Q96QF0	Rab−3A-interacting protein (RAB3IP)	0.044090403	1.51	Cytoplasm/Nucleus
P22570	NADPH:adrenodoxin oxidoreductase, mitochondrial (FDXR)	0.000324249	1.52	Mitochondrion inner membrane
Q8TBQ9	Protein kish-A (TMEM167A)	0.000897747	1.52	Golgi apparatus membrane
O43602	Neuronal migration protein doublecortin (DCX)	0.0498061	1.53	Cytoplasm
Q6WCQ1	Myosin phosphatase Rho-interacting protein (MPRIP)	0.027659338	1.55	Cytoplasm
Q00978	Interferon regulatory factor 9 (IRF9)	0.048513271	1.65	Cytoplasm/Nucleus
O75534	Cold shock domain-containing protein E1 (CSDE1)	0.003960222	1.68	Cytoplasm
P0C870	Bifunctional peptidase and (3S)-lysyl hydroxylase JMJD7 (JMJD7)	0.033199413	1.72	Nucleus /Cytoplasm
A2RTX5	Threonine–tRNA ligase 2, cytoplasmic (TARSL2)	0.048948148	1.72	Cytoplasm/Nucleus
Q9NQA3	WAS protein family homolog 6 (WASH6P)	0.039948885	1.76	Early endosome membrane/Recycling endosome membrane
Q9BZ23	Pantothenate kinase 2, mitochondrial (PANK2)	0.038373648	1.8	Mitochondrion/Cytoplasm
P22033	Methylmalonyl-CoA mutase, mitochondrial (MMUT)	0.016470242	1.8	Mitochondrion matrix
Q9Y2K5	R3H domain-containing protein 2 (R3HDM2)	0.03788905	1.85	Nucleus
O75694	Nuclear pore complex protein Nup155 (NUP155)	0.043543604	1.86	Nucleus/ Nucleus membrane
Q9H9A6	Leucine-rich repeat-containing protein 40 (LRRC40)	0.041906811	1.93	
Q8WVH0	Complexin−3 (CPLX3)	0.008170562	1.94	Cell junction/Cell membrane
O95671	Probable bifunctional dTTP/UTP pyrophosphatase/methyltransferase protein (ASMTL)	0.045696458	2.02	
Q9HCN4	GPN-loop GTPase 1 (GPN1)	0.009829147	2.04	Cytoplasm/Nucleus
O43272	Proline dehydrogenase 1, mitochondrial (PRODH)	0.048318689	2.05	Mitochondrion matrix
P28062	Proteasome subunit beta type−8 (PSMB8)	0.044239435	2.07	Cytoplasm/Nucleus
Q8IUZ5	5-phosphohydroxy-L-lysine phospho-lyase (PHYKPL)	0.008319298	2.08	Mitochondrion
Q765P7	Protein MTSS 2 (MTSS2)	0.032915851	2.11	Cytoplasm
Q9NYL9	Tropomodulin−3 (TMOD3)	0.015993366	2.12	Cytoplasm
Q9GZT6	Coiled-coil domain-containing protein 90B, mitochondrial (CCDC90B)	0.00860084	2.13	Mitochondrion membrane
Q96CM8	Acyl-CoA synthetase family member 2, mitochondrial (ACSF2)	0.031117458	2.14	Mitochondrion
O96007	Molybdopterin synthase catalytic subunit (MOCS2)	0.001271717	2.23	Cytoplasm
Q9NPH2	Inositol−3-phosphate synthase 1 (ISYNA1)	0.000800685	2.26	Cytoplasm
Q96NX5	Calcium/calmodulin-dependent protein kinase type 1G (CAMK1G)	0.010175008	2.32	Cytoplasm/Golgi apparatus membrane/Cell membrane
Q15751	Probable E3 ubiquitin-protein ligase HERC1 (HERC1)	0.047357798	2.32	Membrane/Cytoplasm
Q9UJV8	Purine-rich element-binding protein gamma (PURG)	0.007938768	2.38	Nucleus
P15559	NAD(P)H dehydrogenase [quinone] 1 (NQO1)	0.03367561	2.4	Cytoplasm
Q9NWY4	Histone PARylation factor 1 (HPF1)	0.044527851	2.55	
P54105	Methylosome subunit pICln (CLNS1A)	0.045372858	2.56	Cytoplasm/Nucleus
O00273	DNA fragmentation factor subunit alpha (DFFA)	0.019867376	2.58	Cytoplasm
Q96BW5	Phosphotriesterase-related protein (PTER)	0.010206613	2.61	
Q8N0W4	Neuroligin−4, X-linked (NLGN4X)	0.032117539	2.77	Cell membrane
A4D1E9	GTP-binding protein 10 (GTPBP10)	0.034690156	2.78	Nucleus
P51161	Gastrotropin (FABP6)	0.042109455	2.8	Cytoplasm/Membrane
P80294	Metallothionein−1H (MT1H)	0.046577618	2.82	
Q5TC84	Opioid growth factor receptor-like protein 1 (OGFRL1)	0.021290933	2.83	
Q14244	Ensconsin (MAP7)	0.006018624	2.9	Cytoplasm
Q6UN15	Pre-mRNA 3'-end-processing factor FIP1 (FIP1L1)	0.049883268	2.94	Nucleus
Q5VZK9	F-actin-uncapping protein LRRC16A (CARMIL1)	0.003988942	3.02	Cytoplasm/Cell membrane
O95210	Starch-binding domain-containing protein 1 (STBD1)	0.034371792	3.11	Preautophagosomal structure membrane/Endoplasmic reticulum membrane
Q96EL3	39S ribosomal protein L53, mitochondrial (MRPL53)	0.04263729	3.11	Mitochondrion
Q8N8A2	Serine/threonine-protein phosphatase 6 regulatory ankyrin repeat subunit B (ANKRD44)	0.016068682	3.54	
Q9NPR2	Semaphorin−4B (SEMA4B)	0.009761574	4.32	Membrane
Q8WV93	AFG1-like ATPase (AFG1L)	0.004871979	5.03	Mitochondrion
P04259	Keratin, type II cytoskeletal 6B (KRT6B)	0.043797443	5.29	Cytoskeleton/Cytosol/Extracellular region or secreted
O75881	25-hydroxycholesterol 7-alpha-hydroxylase (CYP7B1)	0.044656159	5.3	Endoplasmic reticulum
Q9UL12	Sarcosine dehydrogenase, mitochondrial (SARDH)	0.002488866	5.73	Mitochondrion
Q9BXV9	EKC/KEOPS complex subunit GON7 (GON7)	0.011582219	6.03	Nucleus
Q7Z449	Cytochrome P450 2U1 (CYP2U1)	0.018037731	8.15	Endoplasmic reticulum
Q92692	Nectin−2 (NECTIN2)	0.013976793	8.63	Plasma membrane
O15231	Zinc finger protein 185 (ZNF185)	0.014334596	10.77	Cytoskeleton/other locations

**Figure 7:**
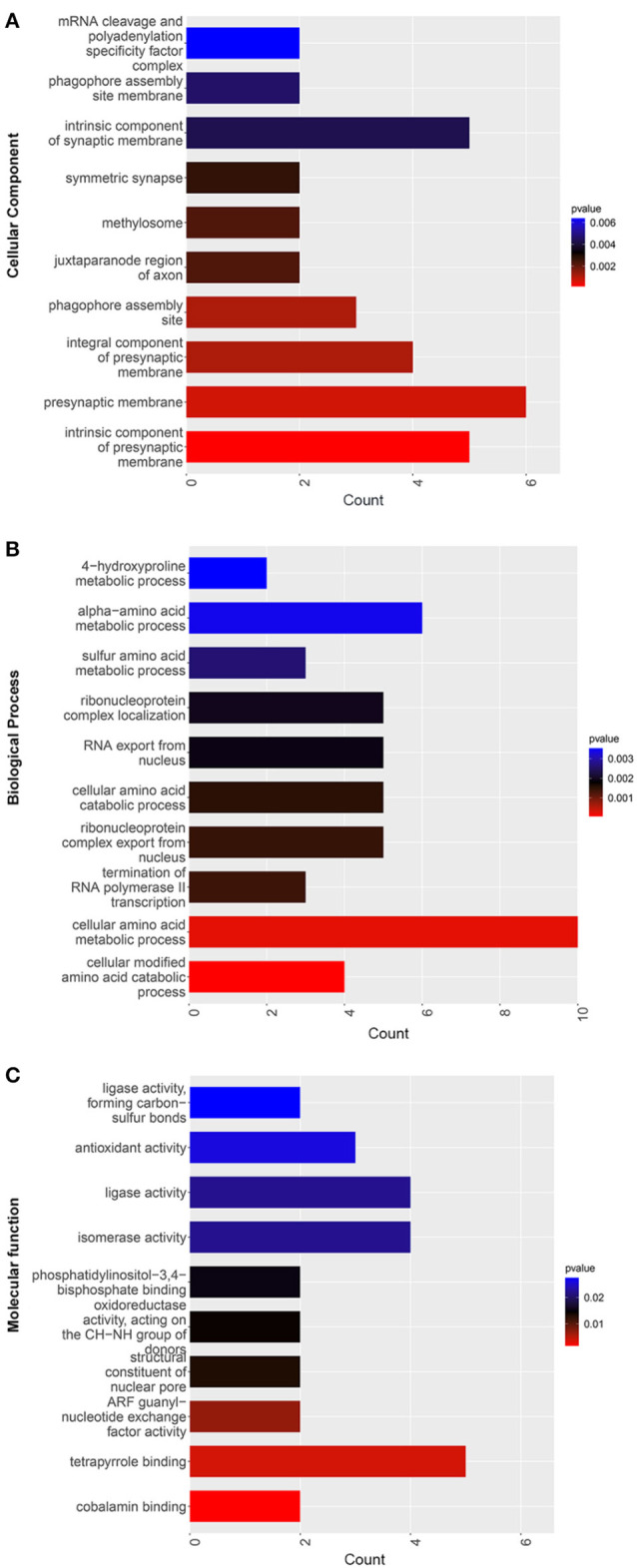
GO enrichment map of differentially expressed proteins between the TSC1 truncating mutation group and the control group. The y-axis represents the classification of differentially expressed proteins according to **(A)** Gene Ontology Cellular Component (GO CC), **(B)** Gene Ontology Biological Process (GO BP), and **(C)** Gene Ontology Molecular function (GO MF). The x-axis is the count of differentially expressed proteins.

The GO CC enrichment analysis of the differentially expressed proteins in the TSC1 truncating mutation group and the control group can provide a reference for the analysis of the enrichment location of the differentially expressed proteins in the cells. According to the analysis results, the differentially expressed proteins were mainly enriched in the mRNA cleavage and polyadenylation specificity factor complex, the phagophore assembly site membrane, the intrinsic component of the synaptic membrane, the symmetric synapse, the methylosome, the juxtaparanode region of axon, the presynaptic membrane, the phagophore assembly site, the integral component of the presynaptic membrane, the intrinsic component of the presynaptic membrane and so on (Figure 7A). It is worth noting that there is a considerable amount of differentially expressed protein enrichment in the intrinsic component of the synaptic membrane and the presynaptic membrane, indicating that the normal function of the synapse has undergone substantial changes.

The GO BP enrichment analysis of the differentially expressed proteins in the TSC1 truncating mutation group and the control group can provide a reference for analyzing the biological processes involved in the differentially expressed proteins. According to the results of the analysis, differentially expressed proteins are mainly involved in sulfur amino acid metabolic process, alpha-amino acid metabolic process, 4-hydroxyproline metabolic process, termination of RNA polymerase II transcription, RNA export from the nucleus, ribonucleoprotein complex localization, ribonucleoprotein complex export from the nucleus, cellular amino acid catabolic process, cellular modified amino acid catabolic process, etc. (Figure 7B). Among them, the number of differentially expressed proteins involved in cellular amino acid metabolic process is the most, so it may affect cells through the pathway of amino acid metabolism.

The GO MF enrichment analysis of the differentially expressed proteins in the TSC1 truncating mutation group and the control group can be used to summarize and analyze the molecular functions of the differentially expressed proteins in cellular biological activities. According to the results of the analysis, the differentially expressed proteins mainly exert ligase activity, forming carbon-sulfur bonds, antioxidant activity, ligase activity, isomerase activity, phosphatidylinositol-3,4-bisphosphate binding, oxidoreductase activity, acting on the CH-NH group of donors, structural constituent of nuclear pore, ARF guanyl-nucleotide exchange factor activity, tetrapyrrole binding, cobalamin binding, etc. (Figure 7C).

## Discussion

TSC is a genetic heterogeneous disorder caused by mutations in the TSC1 and TSC2 genes. It can lead to intellectual disability, seizures, and autism (23). In this study, the phenotype of three TSC patients was found to be related to their truncating mutations in TSC1. At present, more than 450 disease-causing mutations of TSC1 have been reported (24). The nonsense mutation c.1525C>T (p.Arg509Ter) has been reported in 1997 in *Science* (25). Notably, the nonsense mutation c.1524C>A (p.Tyr508Ter) and the frameshift mutation c.1004delC (p.Thr335AsnfsTer3) have not been previously reported in the PubMed and the Human Gene Mutation Database.

Epilepsy is the most common reason for patients with TSC to seek medical advice (15). Treatment of TRE remains a major challenge, with 72–85% of TSC patients having a history of epilepsy, and ~60% of patients suffering from persistent seizures (3). If the use of two or more AEDs is not effective, surgery for patients with epilepsy should be evaluated as early as possible. In the present study, the three cases of intractable epilepsy with TSC1 truncating mutations benefited from surgery. After the accurate positioning of epileptogenic lesions by VEEG and imaging, they underwent total resection of tubers and related cortex. Two cases had complete seizure freedom (Engel I) in an 8-month follow-up.

In the TSC1 truncating mutation group and the control group, we find that some of the differentially expressed proteins have been reported to be associated with epilepsy, including NQO1 (26), MOCS2 (27), PRODH (28), DCX (29), UBA5 (30), SRP9 (31), ARFGEF2 (32), ATG12 (33), MTR (34), KCNA2 (35), ADAM23 (36), KCNAB2 (37), TSC1 (10), IDS (38), GTF2I (39), and so on. Above all, the expression of TSC1 protein is lower in brain tissues with TSC1 truncating mutation, which may be caused by mutations that shorten the protein. The mutation of TSC1 protein affects the mTOR pathway, and the over-activation of the mTOR pathway will produce deformable neurons, which is called “epileptic generator” in TSC (40).

It is worth noting that the expression levels of K^+^ channel proteins, KCNA2 and KCNAB2, were down-regulated in the TSC1 truncating mutation group compared with the control group, which may be related to the effect of seizures on ion channels in patients with TSC. In the context of epilepsy, the K^+^ channel has special significance and is the main determinant of membrane excitability. Native K^+^ currents are produced by the voltage-dependent channel, a tetramer of alfa- and beta-subunits (41). The KCNAB2 gene encodes voltage-gated K^+^ channel beta-subunit, and the decrease of its expression will reduce membrane repolarization, leading to a reduction of the threshold of epilepsy (37). The KCNA2 gene encodes the voltage-gated K^+^ channel Kv1.2, and the mutations in it can cause progressive myoclonus epilepsy and epileptic encephalopathy (35). Additionally, the voltage-gated potassium channel Kv1.1/1.2-subunit is related to cell adhesion molecules (CAM), which includes Caspr2 and LGI1 (42). The interaction of LGI1 with ADAM22 and ADAM23 makes an important impact on the molecular mechanism of autosomal dominant lateral temporal lobe epilepsy (ADLTE). We found that the expression level of ADAM23 decreased in the TSC1 truncating mutation group. Some studies have shown that ADAM23 gene is associated with epilepsy in mice and dogs. However, the potential value of ADAM23 in the clinical is still unclear, so clinical studies are needed to explore whether ADAM23 protein is related to the incidence of epilepsy patients (36).

In addition to the genes related to epilepsy, there are also some genes related to nerve migration. Among the differentially expressed proteins, we detected a decrease in the expression level of ARFGEF2. Some studies have shown that ARFGEF2 mutations can lead to severe intellectual disability and are closely related to neuronal migration disorders. However, the molecular mechanism of the neuronal migration regulated by BIG2 (encoded by the ARFGEF2 gene) is not clear, and further study is needed (32). Tubers are epileptogenic cortical malformations in TSC. Allana et al. showed that both mRNA and protein levels of the DCX protein were higher in TSC nodules than in the control group (43). This result is consistent with our expression of differentially expressed proteins. DCX is related to neuron migration, and the expression of DCX in the lesion area of patients with tuberous sclerosis is higher than the normal control, which may be related to abnormal neuronal localization (heterotopia), intellectual disability and epilepsy caused by DCX (29).

Additionally, the expression of ATG12, a kind of autophagy-related protein, is reduced in the TSC1 truncating mutation group. Some studies have pointed out that autophagy of hippocampal neurons after induced seizures may be a new idea for the treatment of epilepsy. As far as we know, autophagy is strictly regulated by autophagy-related molecules (collectively referred to as ATGs). Therefore, it may be a new therapeutic idea by regulating the expression of ATG12 protein to reduce seizures (33).

We also compared the results of differentially expressed proteins with previous studies about gene expression profiles in TSC mice or patients. Compared to transcriptomic data (DEG) and proteomic data (DEP) of forebrains from 3 Tsc1/Emx1-Cre mice and 3 wild-type mice, transcriptomic data of cortical tubers from 4 TSC patients and 4 autopsy control tissues (GSE16969), and transcriptomic data of brain tissues from 3 TSC patients and 3 normal brain tissues (GSE62019) (44), there are 6 up- and 8 down-regulated proteins in our present study that overlap with these four databases (Table 4). The trends of differentially expressed proteins including TSC1, PRODH, CSDE1, BUB3, and PRPSAP2 in our study are consistent with those in mice databases (DEG and DEP). Also, the trends of differentially expressed proteins including RAB3IP, ACSF2, CYP2U1, TNFRSF21, KCNAB2, COPZ1, PRDX3, FDXR, and KLKB1 in our study are consistent with those in human databases (GSE16969 and GSE62019).

**Table 4:** Differentially expressed proteins that overlap with the databases.

	**DEG**	**DEP**	**GSE16969**	**GSE62019**
Up	PRODH	CSDE1	RAB3IP ACSF2 CYP2U1	FDXR
Down	TSC1	BUB3 PRPSAP2	TNFRSF21 KCNAB2 COPZ1 PRDX3	KLKB1

*DEG, transcriptomic data of forebrains from 3 Tsc1/Emx1-Cre mice and 3 wild-type mice; DEP, proteomic data of forebrains from 3 Tsc1/Emx1-Cre mice and 3 wild-type mice; GSE16969, transcriptomic data of cortical tubers from 4 TSC patients and 4 autopsy control tissues; GSE62019, transcriptomic data of brain tissues from 3 TSC patients and 3 normal brain tissues*.

Through the GO enrichment analysis of TCS mutation and control differentially expressed protein, we found something interesting. Firstly, in the classification of cell structure, the differentially expressed proteins are mainly concentrated in the intrinsic component of synaptic membrane, presynaptic membrane and so on. This suggests that the TSC1 truncating mutation may cause changes in the normal function of synapses, thus affecting the transmission of information between cells. Secondly, in the classification of biological processes, differentially expressed proteins are mainly enriched in the process of amino acid metabolism. There may be two main reasons for abnormal amino acid metabolism, one is the enzyme defect, which blocks the amino acid catabolism, and the other is the defect of amino acid absorption and transport system. Additionally, in the classification of molecular function, the differentially expressed proteins are mainly enriched in antioxidant activity, ligase activity, isomerase activity, tetrapyrrole binding. It is worth noting that tetrapyrrole binding has a large number of differentially expressed protein enrichment.

Our study provides a new idea to explore the brain injury mechanism caused by TSC1 mutations. Unfortunately, due to the difficulty of clinical sample collection, the sample size of this study is not large enough. The results of differentially expressed proteins need to be further verified in a large sample study. Moreover, if the difference of protein expressions in the brain between patients with TRE and patients with non-TSC epilepsy can be analyzed, it may be helpful to explore the unique pathogenesis of TRE.

## Conclusion

To sum up, the study investigated clinical features, mutation analyses and brain proteomic profiling of three surgical cases of intractable epilepsy caused by truncating mutations in the TSC1 gene. In this study, we reported two new mutations in the TSC1 gene, expanding the mutation spectrum of the TSC1 gene related to clinical phenotype. Proteomic profiling suggested that the differentially expressed proteins were mostly concentrated in the synaptic membrane between the patients with TSC1 truncating mutation and the control group, indicating that the changes of synaptic membrane in patients with TSC may affect the information transmission between cells. Amino acid metabolism is also significant among differentially expressed proteins, highlighting the probable key role of abnormal amino acid metabolism in the mechanism of brain damage caused by TSC1 mutations.

## Data Availability Statement

The datasets presented in this article are not readily available because of legal restrictions. Requests to access the datasets should be directed to the corresponding author Qing-Xia Kong.

## Ethics Statement

Written informed consent was obtained from the individual(s), and minor(s)' legal guardian/next of kin, for the publication of any potentially identifiable images or data included in this article.

## Author Contributions

Q-XK, J-CZ, and Q-BL designed the present study. X-BH, HY, H-XY, J-HF, LW, HZ, and BZ collected the clinical data. Y-DL and M-YM analyzed the data and wrote the paper. Y-KZ revised the paper. All authors read and approved the final version of the manuscript.

## Conflict of Interest

The authors declare that the research was conducted in the absence of any commercial or financial relationships that could be construed as a potential conflict of interest.

## Footnotes

^1^http://ncbi36.genepipe.ncgm.sinica.edu.tw/primerz/beginDesign.do

^2^http://bioinf.cs.ucl.ac.uk/psipred/

^3^https://cran.r-project.org/src/contrib/Archive/ggplot2/

## Supplementary Material

Supplementary Figure 1 The VEEG of case 1. **(A)** There are medium-high amplitude sharp waves and spike-and-wave complexes in the left frontal, middle and temporal regions. **(B)** Spike-and-wave complexes of high amplitude and extremely high amplitude occur in all regions.

Supplementary Figure 2 Sanger sequencing of candidate sites in the TSC1 gene of the three patients and their families. The heterozygous mutation c.1524C>A in **(A)** case 1, and wild-type sequence in **(B)** the father and in **(C)** the mother. The heterozygous mutation c.1525C>T in **(D)** case 2 and in **(E)** the mother, and **(F)** wild-type sequence in the brother. The heterozygous mutation c.1004delC in **(G)** case 3 and in **(H)** the father, and **(I)** wild-type sequence in the mother.

Supplementary Table 1 The information of the control group.
