# Supplementary figures and images for: Brain Proteomic Profiling in Intractable Epilepsy Caused by TSC1 Truncating Mutations: A Small Sample Study
Source: Front Neurol. 2020 May 29;11:475. doi: 10.3389/fneur.2020.00475 (PMC7326032; doi:10.3389/fneur.2020.00475)

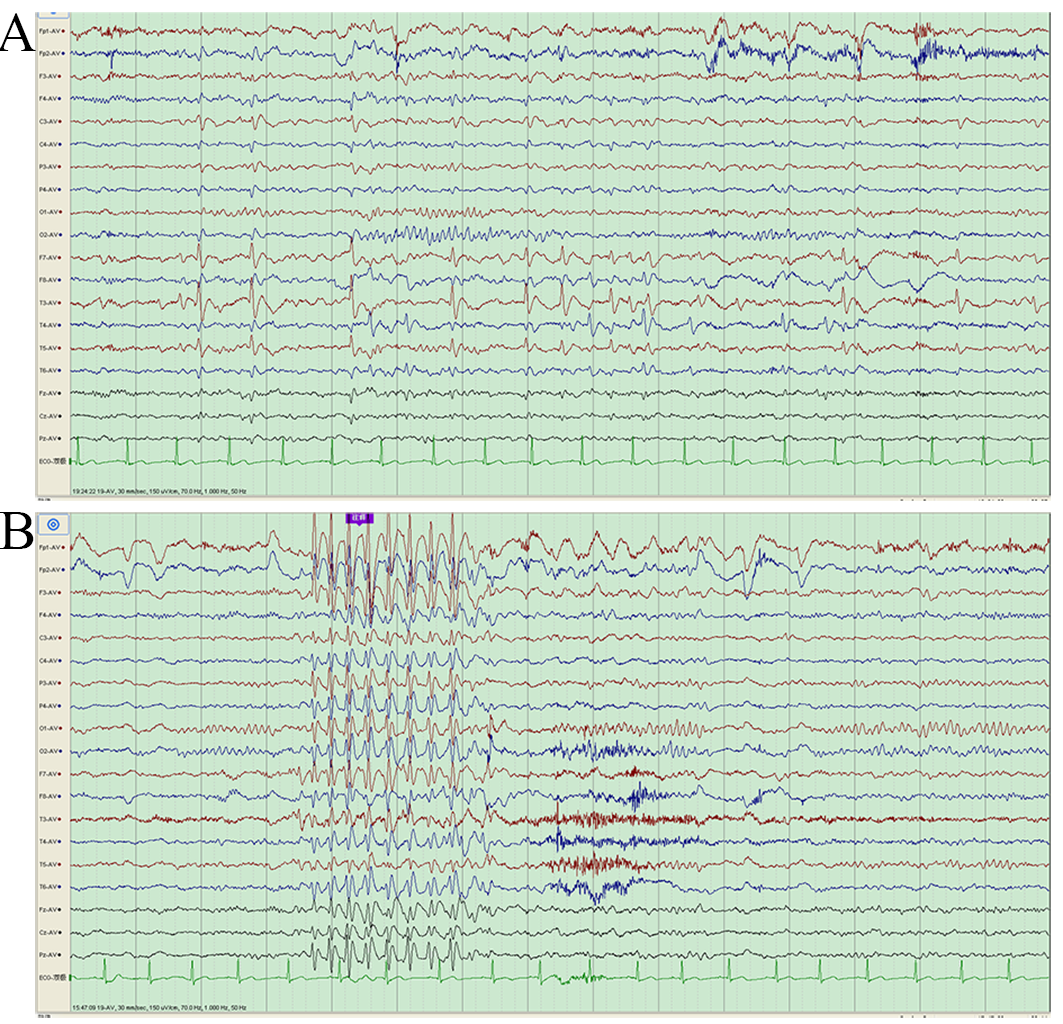

Supplement: Supplementary Figure 1 — The VEEG of case 1. (A) There are medium-high amplitude sharp waves and spike-and-wave complexes in the left frontal, middle and temporal regions. (B) Spike-and-wave complexes of high amplitude and extremely high amplitude occur in all regions. [file Image_1.TIF]

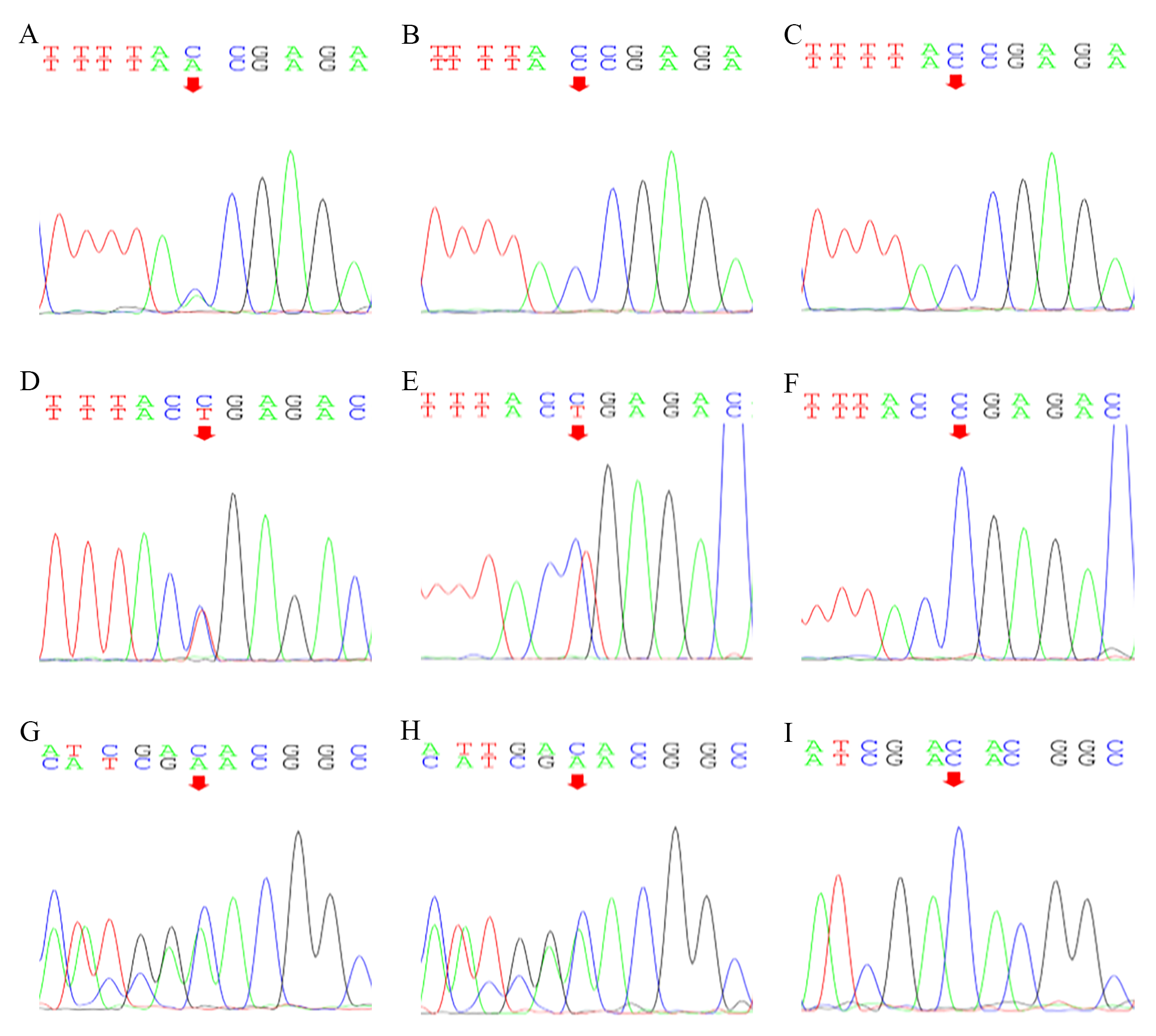

Supplement: Supplementary Figure 2 — Sanger sequencing of candidate sites in the TSC1 gene of the three patients and their families. The heterozygous mutation c.1524C>A in (A) case 1, and wild-type sequence in (B) the father and in (C) the mother. The heterozygous mutation c.1525C>T in (D) case 2 and in (E) the mother, and (F) wild-type sequence in the brother. The heterozygous mutation c.1004delC in (G) case 3 and in (H) the father, and (I) wild-type sequence in the mother. [file Image_2.TIF]
