# Supplementary material for: Brain Proteomic Profiling in Intractable Epilepsy Caused by TSC1 Truncating Mutations: A Small Sample Study
Source: Front Neurol. 2020 May 29;11:475. doi: 10.3389/fneur.2020.00475 (PMC7326032; doi:10.3389/fneur.2020.00475)
Supplement: Supplementary Table 1 — The information of the control group. [file Table_1.DOCX]

Supplementary Table 1 | The information of the control group.

|  | Age  (year) | Gender | Diagnosis | Surgical site | Epilepsy history | Family history of epilepsy |
| --- | --- | --- | --- | --- | --- | --- |
| Control 1 | 13 | Male | Craniocerebral trauma | The left frontal lobe | No | No |
| Control 2 | 21 | Male | Craniocerebral trauma | The left temporal lobe | No | No |
| Control 3 | 10 | Male | Craniocerebral trauma | The right frontal lobe | No | No |
